# Reviews and Book Notices

**Published:** 1882-10

**Authors:** 


### Reviews and Book Notices.

---

ARTICLE VIII.—THE PRACTICE OF MEDICINE AND SURGERY, APPLIED TO THE DISEASES AND ACCIDENTS INCIDENT TO WOMEN. By WM. H. BYFORD, A.M., M.D., Professor of Gynæcology in the Rush Medical College, etc. Third Edition, thoroughly revised and re-written, with 164 Illustrations. Philadelphia: Lindsay & Blakiston. 1881. Pp. 671.

In the preface of the first edition, Dr. Byford says his "object has been to furnish the students and junior members of the pro-profession a concise, yet sufficiently complete, practical and reliable treatise, to meet their wants in everyday practice."

How fully this object was attained in the first and second editions, may be judged by the verdict of the profession, for the second was soon exhausted, and has been out of print for several years, and I am sure a similar result will follow with the present edition, unless the edition has been unusually large. Of the changes of opinion and practice in this, as compared with former editions, he says: "The progress of medical science in the last decade, especially of that department to which this work is devoted, has made so many changes necessary that the present edition is almost a new book;" and he explains further by saying "that, surrounded as I have been by such a throng of active workers, the results of whose labors I have tried to assimilate, my former ideas have been necessarily greatly modified, and, I hope, improved also."

This frankness and modesty of the preface will be found to be characteristic of the whole work. The work of other writers in all parts of the world is carefully accredited to them, but his own is so modestly stated that one has to search diligently to determine which parts of the work are original with the author—a fact in marked contrast with most writers ; indeed, about the only way to determine which portions of the work are original is by the fact that they are not credited to some one else.

In the first five chapters, the author treats of the diseases of the pelvic organs in their anatomical order; the labia, the perineum, the vulva, the bladder and the vagina.

In rupture of the perineum, while he recognizes the possibility of a spontaneous cure under the most favorable conditions, he advises the immediate closure of the laceration by silver-wire sutures; should this fail, then the secondary operation, when the patient is in good health, and the tissues in condition to heal ; and if they are not so, we must wait till they are before we operate.

In chronic cystitis, he recommends the rapid dilatation of the urethra under ether, instead of the artificial vesico-vaginal fistula as proposed by Dr. Sims, and practiced still by Dr. Emmet, as the simpler procedure, equally safe, and not requiring a second operation to close the fistula.

The importance of puerperal vaginitis is fully insisted upon. The causes producing it, and its effects in the production of vesico-vaginal, recto-vaginal and the other fistulæ, are carefully stated, and should be read and remembered by every physician called to attend a case of confinement. If the instructions here laid down for the prevention and treatment of puerperal vaginitis were carefully followed, there would be few fistulæ to be closed by operation.

The original method of operating upon fistulæ by Dr. Marion Sims, and the modifications of Drs. Emmett, Bozeman and Simon are fully described and amply illustrated, both as to the style of the operation and the instruments required, as well as the preliminary and after treatment of the patient.

Next follow four chapters on menstruation, its disorders and modifications, including puberty, menorrhagia, dysmenorrhœa, hematocele and menopause. The chapter on puberty should be carefully studied by every physician, teacher and mother in the land, that they may fully instruct those committed to their care as to their exercise, associates, dress, education and amusements, during this most important transitional period from girlhood to womanhood. For there can be no question that many of the most intractable and almost incurable cases of displacement, chronic cellulitis, and chronic peritonitis can be directly traced to this period and to the errors committed then for their origin, and that lifelong sterility, or death at the first confinement, are the frequent termination of diseases begun at puberty.

Under the topic menorrhagia, the author incidentally describes the attainments the gynæcologist should possess. It would be well if all who aspire to this title would ponder these sentences, pp. 136-7: It will not be regarded as making an undue claim to say that the practice of gynæcology requires more thorough theoretical and practical familiarity with the details of all the branches of medicine than any of the so-called specialties. We are not prepared to treat the most common of female diseases without being able to scan with scientific scrutiny every organ and function of the body. For not until we can compete successfully with the general practitioner, the surgeon, the clinist and the neurologist in the therapeutic processes of their respective departments, may we hope to exercise, in the highest sense, the office of the gynæcologist.

In the treatment of dysmenorrhœa caused by stenosis, while the author seems to advocate the method of dilatation by the use of the slippery elm or laminaria tent, or the sponge tent as directed by Dr. Storer, yet he speaks first of the method by incision, and describes in minute detail the operation of Dr. Sims and the modifications of Dr. Peaslee, without comment, and leaves the reader in doubt as to which method is to be preferred, or how the cases are to be selected for each plan of treatment.

Metatithmenia, or misplaced menstruation and peri-uterine hematocele are treated as synonymous, and vicarious menstruation is not mentioned.

Menopause is not considered, as Dr. Tilt thinks, the cause of many diseases, though it is contemporaneous with a number of the most dangerous affections; still, we need not fear that the change of life will be disastrous, either as a cause of disease, or by injuriously modifying those already existing.

In chapter eleven, acute inflammation of the unimpregnated uterus is insisted upon, as of other organs, in marked contrast with some recent writers on gynæcology, who teach that there may be congestion of the unimpregnated uterus, but that it can never, or very rarely, go so far as inflammation of the deeper structure of the uterus, but affects only the mucous membrane.

In chapters twelve to eighteen we have perhaps the most important part of the work to the general practitioner, treating of the reflex and local symptoms of uterine disease, their pathology, etiology and diagnosis, with the general and local treatment. The chapters on the reflex and local symptoms are especially full and complete, and no intelligent physician, having fully studied these chapters, and then examined a considerable number of patients presenting these symptoms, as directed in the chapter on diagnosis, and adopted the general and local treatment here advised for a few months, could doubt that the sexual system of the female, in the state of disease, exercises a very morbid influence over nearly the whole organism, and that the only sure and permanent relief is found in the cure of the diseased condition of the sexual organs.

But these chapters cannot be described; they must be studied to be appreciated.

To Dr. Emmet, says the author, belongs the credit of first appreciating the importance of and appropriately treating laceration of the cervix. "It very seldom occurs to any man to have the opportunity of giving the profession so complete a description of the abnormal condition, and to perfect the process of cure, so that there is left to others no room for improvement;" a compliment well deserved and truthful, and yet in marked contrast to Dr. Emmet's book, in which he "aims to represent the actual state of gynæcological science and art," but does not allude to Dr. Byford as writer or worker in the field.

Dr. Byford alludes to cellulitis and local peritonitis as complications of laceration of the cervix, but does not speak definitely of the presence of these diseases as contraindications for the operation, though such surely is his practice.

A disregard of these contraindications has indirectly been one of the causes of the fatal result which occasionally follows this operation, and explains why some patients are injured rather than benefited by the operation. It would have been well to have repeated the cautions given in displacements of the uterus. "It should be remembered that these adhesions are the result of inflammation, and generally local peritonitis, and that this last-named condition is often present in a subdued degree for a long time after the effusion resulting from it has hardened into a false membrane. This circumstance will be a standing caution to the considerate practitioner. It will be easy, by a little indiscretion in our manipulation, to arouse a more acute form of inflammation, and thus do much mischief.

"When there is tenderness behind the uterus, we cannot be too careful; indeed, we should always wait when there is good reason to apprehend local peritonitis, until every evidence of it has subsided, and this will generally require a long time, even if judicious treatment is employed."

And again, in speaking of replacing the retroverted uterus with a lacerated cervix and shortening of one of the broad ligaments, he says: "In all such cases, there is good reason to fear that there is a lurking chronic inflammation in the ligament to which the cervix is attached, which upon slight provocation will be aggravated and become acute."

The chapters on perimetritis and chronic perimetritis need to be carefully studied and their teaching remembered in operating upon the lacerated cervix and treating the displaced uterus.

Our author states his creed in regard to displacements in a a single sentence, and it should be remembered. "I believe that pre-existing disease is the cause of displacements of the uterus in the great majority of instances, and that the displacement is very seldom the cause of the disease usually associated with it."

The cautions in regard to the use of pessaries should be read, re-read, and always recollected. "Hyperæsthesia from inflammation of the uterus, especially in the acute and subacute forms, is incompatible with the use of pessaries. Immobility of the uterus from old adhesions must be be overcome before we can venture to use pessaries. Perimetritic inflammation in the acute, subacute and chronic forms, contraindicates the use of support." In all cases, the instrument should not only not cause pain, but should be attended with a sense of relief.

The causes of that rare but dreadful accident, inversion of the uterus, are fully stated, and should be carefully studied by every accoucheur, for the inference from statistics is that the accident is nearly, if not quite, avoidable, for no cases were observed either by Dr. West or Dr. McClintock in the London Maternity, Charity, or the Dublin Lying-in Hospital—140,000 cases.

Two plans of treatment of the chronic form are described; the one by Dr. White, of Buffalo, and the other by Dr. Tyler Smith, of London.

Dr. White's repositor in use is figured, but though Dr. Tyler Smith's method is preferred and used by the author, neither the instrument nor the manner of its use are figured. There is, it is true, a full description of the manner of using the elastic water bag and its action in reducing the inverted uterus, still, a figure would as much aid the student and the physician who are not used to the instrument, as it does with Dr. White's instrument. "The fact cannot be too forcibly impressed upon our minds, in undertaking this operation, that gentle firmness is the proper expression for the force to be employed. Perseverance, instead of violence, is both more certain, successful and secure, in overcoming the resistance of muscular fiber anywhere, and this is especially true with the uterus, the strongest muscle in the body."

In cases where restoration is proven impossible, amputation is the last resort, and the methods are described, preference being given to the galvano-cautery.

The chapter on diseased deviations of involutions of the uterus is one of the most complete and satisfactory in the whole work. It is divided into the topics, 1. Delayed involution, 2. Subinvolution, and 3. Hyperinvolution.

The term involution is applied also to the uterus at the menstrual period, the changes recuring before or after the menstrual act being analagous to the evolution and involution of pregnancy, though of course less in degree.

"The term and process of involution extend to the changes observed—all the genital organs, the lacteal glands, the ovaries, uterus, vagina, Fallopian tubes, uterine ligaments and perineum."

And the importance of subinvolution is not exaggerated in the statement "that more of the chronic congestions of the uterus originate in the puerperal and menstrual subinvolution, as here explained, than in any other condition."

The treatment is described as preventive and local.

"During labor, everything should be conducted with the view of preserving the integrity of the soft parts, because damage to any of the parts concerned in labor is pretty sure to be followed by subinvolution.

"The more physiological a labor is made, and the more skilfully conducted, the less the tendency to subinvolution. After labor, complete contraction should be brought about and maintained, not by mechanical irritation, but, if need be, by the use of ergot and vaginal injections of hot water. The most assiduous attention should be especially given to control all inflammations that follow labor.

"More attention and care in conducting patients through cases of abortion and premature labor should be practiced than is usual.

"In chronic cases, the local treatment is of prime importance. If there is laceration of the cervix or perineum, it should receive attention; if misplacement, it must be corrected.

"The use of glycerine tampons and hot water injections will be found applicable and beneficial in most cases. But there is another class of local remedies that I believe to be more serviceable than these, and that is local stimulants applied directly to the mucous membrane, such as iodine, carbolic acid, tr. of iron, etc. The vaso-motor nerve supply of the whole uterus is so intimately connected that it may be considered a unit, and no part of it can be stimulated without affecting the whole."

Cancer is described as of four varieties. 1. Medullary. 2. Epithelial. 3. Colloid. 4. Scirrhus; though the two latter are exceedingly rare. Of the prognosis of cancer he truthfully says, "it is a gloomy one. Indeed, there is no disease which so uniformly terminates fatally as cancer of the uterus. But the gloomy picture is in part relieved by the improved palliative means we now possess."

Of the many medicinal agents for a time enjoying the reputation of cancer cures, such as jaborandi, cundarango, etc., all but one are passed by in disappointment. To this one, chian turpentine, perhaps because it is the last, our author surely gives more space than it justly deserves, devoting seven pages in small type to Prof. Clay's report of the successful use of this remedy in four cases, with other cases under treatment showing similar effects, and still other cases of cancer in other organs, with remarkable benefit.

But those who have used the chian turpentine in this country have been disappointed in its use, and even the turpentine furnished by Dr. Clay himself, and given according to his written directions, in one case, did not arrest the disease, but the patient went on rapidly to death.

While our author does not fully recommend the formidable operation of total extirpation of the uterus, he "yet hopes that some method of resecting it will some day be invented, which will be less difficult of performance and less dangerous in its results," and he commends highly the operation of Prof. Lane, of California, for removing the uterus by the vaginal method.

Three chapters are devoted to the description and treatment of fibrous tumors of the uterus. They are described as homologous growths of two varieties, the firmer and softer, or myoma and fibroma, but probably both should be named myoma, as they each consist of undeveloped muscular tissue.

Chronic hyperæmia seems to be the condition most likely to favor the formation of these tumors, and a case is described in which it seems to have been the cause. The location of these tumors is fully described, and the symptoms they present during their development graphically narrated, as well as their complications of pelvic inflammations, such as peritonitis, cellulitis, etc.

By the adhesions produced by these local inflammations, new sources of vascular supply are acquired, and new vitality at once imparted to them. Subserous tumors not infrequently in this way take on remarkable activity, after having been stationary in their growth for some time.

The complication of pregnancy occurring with these tumors is fully described, and the dangers pointed out; as miscarriage, obstruction in delivery, and incomplete contraction of the uterus, allowing hæmorrhage. The effect upon the tumor is also noted; namely, its complete removal, in not a few cases, during the involution of the uterus following parturition.

The treatment is naturally divided into two divisions, the treatment of the symptoms and the radical cure of the disease, though the means employed for the one purpose may be applied at times also for the other as well.

As the mechanical pressure of these tumors upon the pelvic viscera is a source of great annoyance to the patient, and at times even of danger, it is important to notice the author's directions in such cases; to elevate the tumor before strong adhesions are formed in the pelvis.

"This may sometimes be done by placing the patient in the knee-elbow position and opening the vagina by two fingers, and then pressing the growth upward. The powerful influence of the atmospheric pressure called to our aid by the position and opening of the vagina, is a very material aid in the process of elevation. If this is not sufficient, we may pass the fingers into the rectum, and thus elevate the tumor."

In the medical treatment of these tumors, but a single remedy is recommended; ergot, "and for this remedy it is claimed, 1, that when properly administered, it frequently very greatly ameliorates some of the troublesome and even dangerous symptoms of fibrous tumors of the uterus, e. g., hæmorrhage and copious leucorrhœa; 2, it often arrests their growth and checks hæmorrhage; 3, in many instances it causes the absorption of the tumor; occasionally without giving the patient any inconvenience. 4. By inducing uterine contraction, it causes the expulsion of the polypoid variety. 5. In the same way, it causes the disruption and discharge of the submucous tumor."

A summary is given of 101 cases in which ergot had been used, by 23 different physicians. Of these 22 cases are reported as cured; 39 tumors diminished in size and hæmorrhage stopped; 19, hæmorrhage relieved, but tumor not affected; 21, no result. Such results as these surely warrant a still more extended use of the remedy, and we doubt not a more extensive experience will corroborate these results. The modes of giving ergot vary with different patients and different physicians; some preferring to use it hypodermically; others by the stomach; others still by the rectum; while some will use it by two or more of these methods at the same or different times. But the result is the same, whatever the method of exhibiting the remedy; "and it should be given in the way in which it is, at the time, the best borne by the patient, and in doses sufficient to produce its physiological effect.

"The failure to get any good results in the hands of some practitioners must be attributed to the use of a drug of poor quality, or insufficient quantity, or for too short a time. The treatment of some of my most successful cases extended over many months" says our author.

"The beneficial action of the drug will depend upon the degree of development of the muscular fibers of the uterus and the position of the tumor with reference to the serous or mucous surfaces. The nearer the mucous surface, the better the effects. We can often," he says, "select the cases in which good results may be expected. There are four conditions which are usually reliable for this purpose. They are smoothness of contour, hæmorrhage, lengthened uterine cavity, and elasticity. A smooth, round tumor denotes, for the most part, uniform textural development; hæmorrhage, a certain proximity to the mucous membrane; a lengthened cavity, great increase in the length and strength of the fibers; and elasticity assures us of the fact that cartilaginoid or calcareous degeneration has not begun in the tumor.

"An uneven, nodulated tumor may be composed of many separate solid masses. These displace and prevent the growth of the fibers to such an extent as to render contractions inefficient.

"When hæmorrhage is not present, the tumor is probably near the serous surface, and consequently not surrounded by fibers.

"A short cavity denotes short undeveloped fibers, while hardness is indicative of unimpressible induration. Although I have had no experience in the use of ergot in such cases, I should expect large fibrous cystic tumors to resist its action.

"From this view of the subject it will be seen that I freely admit that there is a large number of cases in which ergot cannot produce any good results, in consequence of their nature, but there is another reason of equal moment why ergot may fail to act upon such cases as would seem to be favorable, on account of the worthlessness of the drug and its preparations."

"When all these causes of failure are considered, the variety of experience met with in the reports upon its trial in the treatment of these tumors is not surprising. It should not, however, be discouraging, but prompt us to more care in selecting the cases and securing reliable preparations of ergot. I have implicit faith in the action of ergot when all the conditions I have pointed out are present. I do not believe it to be uncertain in its action. In addition to the above conditions, I believe perseverance an indispensable condition to success, as it often requires several months to get the best results.

"In concluding," he says, "I desire to disclaim any expectation that ergot will supplant other modes of treatment. The expert surgeon will, as he always has, use his instruments to the neglect of remedies less summary in their effects, and in his hands the maximum of safety will obtain, but there are very few general practitioners who ought, or would be willing to undertake the enucleation of fibroid tumors of the uterus.

"I do claim, however," he says, that the judicious gynæcologist "will lose fewer patients, and secure more cures, by the administration of this medicine, than can be looked for from surgery. I am not surprised that others who have written upon the subject should be incredulous as to the effect of ergot, and the way I account for it is, what I think I can see in their practice as related by themselves, that they do not give the remedy a fair trial. They fail to give it in large enough doses, and do not persevere long enough in its use. The treatment of some of my most successful cases extended over many months.

"When the pains that indicate efficient action, and always precede disruption and expulsion, occur, the practitioner generally becomes alarmed, gives anodynes, and withdraws the medicine, abandoning the case and declaring that ergot is a dangerous remedy. If he had witnessed the same, or even severer pains in labor, he would have encouraged them, and so he should do in expelling the tumor, and the result would be a safe delivery. The tumor would be expelled and the patient relieved.

"But if," says the author, "ergot acts so powerfully in expelling submucous tumors, is there not danger that it may rupture the capsule of the subserous variety, thus expelling them from the uterine substance into the peritoneal cavity, and endanger the life of the patient by causing peritonitis? But there is a great difference in the influence exerted by the uterine cavity on the two varieties of tumor. In the submucous variety, the whole power of the uterine contractions is exerted toward the tumor, driving it in the direction of the os uteri. When the tumor is subserous, the contractions are from the axis of the tumor; their effect is merely to render it pedunculated, and lessen the vascular supply going to it. The main effect will therefore be to check the rapidity of its growth, or to prevent its further enlargement altogether.

"Another question is, does the long-continued administration of ergot induce the gangrene of the extremities that has been attributed to it? And still another, does it cause inconvenience or danger by affecting seriously the nervous centers? After having given this remedy in frequently repeated and large doses, and observed its effects with great care for a number of months consecutively, I can say that I have not noticed any such consequences, though I am not prepared to assert that there is, and always will be, immunity from such effects. The worst symptoms I have witnessed are the severe and persistent pains, and the apparent inflammation of the uterus and peritoneum, where its action has been excessive. These symptoms have, however, been invariably controlled by proper treatment, and have in no instance proved disastrous. In other cases, when the tumor was slowly disintegrated and expelled, a moderate form of septicæmia has invariably occurred, but this condition has not been sufficiently grave to excite alarm in my mind.

"Whether the observation of the profession at large will or will not bear me out in my earnest belief in the curability of some of these tumors by the means I am now teaching, I do not know; but I am sure there is so much logic in the method that it deserves a more extensive trial than has hitherto been made of it."

Of the treatment of these tumors by electrolysis, he says: "In certain desperate cases this seems to me to be a valuable resource; although in the hands of those brilliant surgeons (Drs. Kimball and Cutter), their mode of performing electrolysis seems not to be attended with the dangers one would expect to follow such free penetration of the abdominal cavity and the galvanic excitement of these growths; most of us would hesitate to follow their example." "They will, doubtless, pursue this mode of treatment sufficiently to test its efficacy and its danger, and thus enable the profession to properly estimate its value." "Possibly it will be found that very much smaller electrodes and a less powerful battery may produce effects sufficient to dissipate these tumors, and at the same time greatly reduce the hazard of the operation."

The surgical treatment of fibrous tumors is described under the subdivisions, "Removal of polypoid tumors, Enucleation, Laparotomy, Laparo-Hysterotomy and Oöphorectomy."

In the removal of polypoid tumors, danger may arise from, "1st. Laceration, contusion, or other damage to the uterus, resulting in hæmorrhage or inflammation; 2d. Incomplete ablation—the remaining portion producing sepeticæmia; 3d. Shock sometimes following protracted efforts at removal. Torsion and amputation are the methods preferred for the removal of polypoid tumors, amputation being performed by the scissors, knife, ecraseur or galvano-cautery wire.

"Hæmorrhage, which was formerly most feared, is now considered of minor importance.

"The removal by torsion is preferred when practicable, as there is no danger from hæmorrhage or septicæmia, as the vessels are torn and no portion of the tumor is left to slough."

If the cervix is not already sufficiently dilated to allow the proper manipulation, it is to be dilated before operating.

"Enucleation consists in splitting the capsule and turning the tumor out of its bed. The first step is thorough dilatation of the cervix, if it is not already sufficiently open to admit the fingers as far up into the cavity of the uterus by the side of the tumor as they can reach. If the vagina is small, it should also be prepared by dilating. An obstacle sometimes encountered is the imperfect separation of the tumor from the other tissues of the uterus by the capsule or the adhesion of the tumor to its capsule. If this condition could be diagnosed beforehand it would contra-indicate the operation for enucleation.

"The danger after the operation is to be apprehended from shock, hæmorrhage, inflammation and septicæmia. But septicæmia can be more effectually treated after this than after almost any of the other great operations, as the cavity of the uterus can be kept clean by hot carbolized injections.

"Laparotomy we may avail ourselves of for the extirpation of these tumors when less hazardous measures are either impracticable or unavailing.

"The incision in the abdominal walls is made in the same place and in the same way as for ovariotomy, though usually it will be necessary to make it much larger.

"If the tumor is pediculated, the pedicle is to be ligated with a double silk ligature, the tumor removed and the stump dropped into the abdomen as in ovariotomy.

"If the tumor is sessile, and the base too broad to be included in a ligature, the capsule should be freely opened and the tumor enucleated. After which, if the capsule is large, provision should be made for drainage through the uterine cavity, and the capsule closed with 'silver wire sutures.'"

We cannot understand why the author prefers silver wire to the silk or animal sutures.

"When the tumor is situated in the posterior wall of the uterus and small, it may be removed through the vagina by an incision through the posterior vaginal wall.

"In laparo-hysterotomy or the removal by abdominal section of both tumor and uterus and perhaps the ovaries also, the operator is to be especially prepared to stop hæmorrhage, as these tumors are very vascular and are liable to have extremely vascular extensions, and the vascular supply of the uterus is enormously increased as in pregnancy."

The application of the elastic bandage to the tumor before its removal, as recommended by Labbè, is especially commended, as by this means we save to the patient a large quantity of blood, which she can ill afford to lose."

"The elastic bandage is important, also, to control hæmorrhage until the vessels can be permanently secured."

The operation is considered "far more grave than ovariotomy, or even double ovariotomy, as by this the whole genital system, with all its reflex capacities and sympathetic relations, is suddenly torn from its connections."

"In operations of this kind, conservative surgery," he says, "is of the greatest importance, and we ought never to remove the ovaries when we can preserve them."

But if we remove the uterus and leave the ovaries, we leave the patient, if she recover from the operation, in the condition of the unfortunate patient with congenital absence of the uterus but with active ovaries.

And it is in this condition—congenital absence of the uterus with active ovaries, that Battey's operation or Oöphorectomy is especially recommended. But the cases are not parallel, for in the congenital case there are not likely to be inflammatory adhesions about the ovaries to tease the patient and produce reflex effects, as there must of necessity be after operations in which the ovaries are left. So that, while removal of the ovaries and uterus renders the operation more hazardous at the time, it obviates the necessity of a subsequent oophorectomy.

Battey's operation, or oöphorectomy, was designed to artificially induce menopause, and in that way, it was hoped, relieve many of the intolerable and irremedial forms of oöphoro-neuroses that so often perplex the practitioner.

"The operation has also been repeatedly performed for the purpose of arresting the growth of fibrous tumors of the uterus, on account of the favorable effect the natural menopause so generally produces upon them, and in some instances with favorable results."

Our author credits Dr. Battey with "removing the ovaries for the purpose of artificially inducing the menopause," and then reminds the reader that menopause is not change of life, and that this condition—menopause—is something brought about by some of the very conditions for which Battey's operation is performed without producing change of life. But Dr. Battey, in a recent private letter, says: "The object sought in my operation is not the menopause, but the 'change of life by art.' It is applicable to cases where the menses are absent, or, in other words, where the menopause has already occurred."

"The mortality of less than 23 per cent in Battey's operation certainly commends it. And if, as Dr. Mundè observes, the positive benefits of the operation were as assured as the favorable rate of mortality, the opposition would soon cease."

Dr. Maunder collected thirty-eight cases of oöphorectomy ; of these twenty-nine recovered from the operation and nine died. And of the twenty-nine who recovered from the operation, twenty-five are reported as cured, two as not cured, and two not accounted for. But one, at least, of the twenty-five reported cured did not remain so—(Dr. Trenholme's case).

Our author very wisely says there is another side to this subject. "The general condition of the patients, who are the subjects of these nervous symptoms, is such as in part to account for their sufferings. And we sometimes find that a radical change in the circumstances under which they live will dispel their trouble. Muscular labor, out-door exercises, the loss of luxuries, when brought by inexorable bad fortune, have done wonders in the way of removing oöphoroneuroses."

Dr. Battey's three questions are repeated : 1st. Is this a grave case ? 2d. Is the case incurable by any other known resource of medical or surgical art ? 3d. Is it curable by the menopause or change of life ? If all these questions are answered in the affirmative, the case is a proper one for Battey's operation ; but if either cannot be answered satisfactorily in the affirmative, the case is one in which the operation is not justifiable.

Both Dr. Battey and our author prefer the abdominal method for operating unless the ovaries are much prolapsed and are certainly free from adhesions and the vagina large.

The results of the author's cases are that the patients are sterile and do not menstruate, but they are not unsexed in any other sense. "In morals, manners, appearances, affections, propensities and voice they remain the same."

Seven chapters are devoted to the diseases of the ovaries and their treatment. In describing the symptoms of ovarian disease, our author wisely calls attention to the fact "that the nerve supply to these organs is essentially a unit, and it is therefore nearly impossible for us to separate the general symptoms arising from the diseases of the pelvic viscera, into vulvar, vaginal, uterine and ovarian, though it is questionable whether all of the hysteroneuroses should not be regarded as oöphoro-neuroses, that is direct or indirect morbid emanations from the ovaries themselves. But in these organs we have a circle of functions, to the perfection of which, soundness in all is essential."

The question is asked, are displacements of the ovaries always necessarily accompanied by serious local or destructive general disturbances; and the reply given, No. But in explaining the answer, a single reason only is given, "the *nervous susceptibility* of the patient, either congenital or acquired."

Now, while it is true that the same nerves are more sensitive in some persons than in others, and in the same person at different times, is not the more important explanation in the fact that ovaries are not always diseased, though displaced, and when they are not, there are few or no symptoms of reflex or local disease? This is also true of the uterus, which may be displaced, but if not diseased itself, and if it has not produced disease in the adjacent organs by its malposition, the patient will not suffer from "uterine symptoms."

As those patients suffering from ovarian diseases are usually anæmic or hydræmic, the indication is to improve both the quantity and the quality of the blood, and in this way improve the functional activity of the nerve centers. "Such cases being usually cases of blood exhaustion, rather than nerve exhaustion, and the treatment consists in turning these patients completely around in their habits and lead them to adopt measures that will make them form plenty of blood and fat.

"Dr. Wier Mitchell has taught us how to do this, and his system of managing such patients is admirable, but it is not al ways practical nor, indeed, necessary to adopt his method as a whole. Absolute rest is necessary only in cases of extreme prostration. In most cases, active exercise will be better than passive, but the exercise should be prescribed in quantity and kind, and enforced with exacting regularity and urged by a decision that will not fail.

"But the most important part of the treatment is the regulation of the food, the prescription of its items and quantity from day to day"—and these are fully stated. "If the food is not rejected by vomiting, or it does not irritate the bowels enough to cause diarrhœa, I would not allow the want of appetite nor the inconvenience that may arise during digestion to be considered a reason for not taking it. Usually the stomach will become tolerant, and after a time the enriched blood, circulating through its glandular apparatus, will engender a relish for food, and the patient will eat with pleasure.

"With this, or some other equivalent method of feeding the patient, there should be associated some plan by which she can get plenty of fresh air, and have as much exercise as she is able to take. The exercise may be passive at first, but as soon as it is possible it ought to be active.

"As long as nutrition can be supplied, the patient will profit by exercise; but if nutrition is impossible, then, of course, exercise is impossible also."

The author belives "this course of management has resulted in a number of instances in averting the dangers and mutilation of the more heroic treatment of castration, by establishing a vigorous and tolerant condition of the nervous system, and thus curing the ovarian irritation. And this plan of treatment is applicable in other cases than displacements of the ovaries, in which there is ovarian irritation.

"In some few cases, when the ovaries are borne down by a displaced uterus, we may occasionally correct the displacement by a well-adjusted pessary, so far as to greatly improve the circulation in these organs, and thus remove a great element in the ovarian distress.

"But in the cases in which the symptoms are most grave, in retroversion and retroflexion of the uterus, the location of the ovaries in the cul-de-sac behind the fundus, renders the satisfactory adjustment of the pessary almost impossible, as the instrument is pretty certain to cause pressure upon these sensitive organs, and thus become intolerant.

"We ought not, however, to despair of accomplishing our object until we have exhausted our ingenuity in mechanical appliances for this purpose.

"And when every other measure fails either to render the condition of the patient bearable or save her from becoming a mental and physical wreck, we still have the resource furnished us by Dr. Battey, of Georgia, namely, the removal of these organs.

"But we should remember that the operation is very dangerous, and that, if successful, it unsexes our patient in the sense that she is at least barren for all future time."

"When the ovaries are displaced so as to occupy the inguinal canal, the operation for removing them is less hazardous, and may therefore be resorted to with less hesitation."

The author relates a single case of simple acute inflammation of the ovaries, the only one he has observed without complications, there being no local peritonitis or cellulitis, and but slight metritis, with the bladder irritable and the vagina slightly tender. The treatment consisted in a simple cathartic, poultices to the abdomen, rest, and anodynes as needed for the pain, and in six or seven days the inflammation was subdued and the patient convalescent, and in three or four weeks was entirely well, and remained so.

Three varieties of ovarian tumor are described; the cystic, fibrous and dermoid, the latter being found in other parts of the body and in the male as well.

The author gives a very full account of the various theories proposed to account for their origin, from the earliest times to the present. The most satisfactory and scientific is, that "at an early period of embryonic development, by some accident or imperfection of formation, an indentation in the blastoderm is produced, and, in the wonderful trophic energy of that period, this minute depression is enclosed by the approximation of its margins, and becomes an isolated cavity, and the growth and perfection of the embryo are accomplished, notwithstanding this early accident to the integrity of its envelope."

But we have not the space to reproduce even a sample of this interesting article, and we must refer the reader to the work itself, where he will find a full report of four cases, and a more satisfactory description of these strange tumors than can be found in any other work to which the English reader has access.

Fibroid tumors of the ovaries are so rare that they receive but a passing notice, but nearly six chapters are devoted to the etiology, prognosis, diagnosis and treatment of the ordinary ovarian tumors—cystomata.

As to the cause of these tumors, our author does not agree with most writers, who "would trace all chronic enlargements to a scrofulous taint, but rather explains their origin by supposing inflammation of a low grade and somewhat chronic duration, causing thickening and induration of the indusium, so that it would not yield to the upheaving pressure of the ovisac and permit its dehiscence. When once thus commenced, the stimulus of increased secretion of fluid would produce a kind of hypertrophy in the involucra, which would permit of a further enlargement. But while inflammation is probably the cause of the beginning of the development of ovarian tumors, it does not seem necessary to their continued development, as the accumulation of fluid in a shut cavity with a secreting external surface is a matter of necessity, and the limit of its amount, for the most part, does not depend upon anything but the capacity of the involucra to grow, until interrupted by external circumstances.

"Prognosis. Unfortunately, there is almost no tendency to spontaneous recovery in ovarian dropsy. Probably not two per cent. but would, after a longer or shorter period, terminate in the death of the patient. But this does not properly represent the value of a life threatened by this affection. Some patients live a great many years in comparative comfort, but generally the case is very different, only a few years finishing its course in a downward direction ; the average duration of life being about three years from the time it is first noticed.

"Our prognosis will depend upon the form of tumor ; whether monocystic or polycystic, the monocystic being much the more favorable for treatment, and more likely to terminate in spontaneous recovery. Again, our prognosis will be influenced by the age of the patient ; occurring in young persons it is more likely to advance rapidly than in old ones. It will advance less rapidly after menstruation ceases than before, and the earlier in menstrual life, the more rapidly will it advance."

Our author is a firm believer in the diagnostic importance of the granular cell. He says: "I have never found the ovarian cell described by Drysdale in any but ovarian fluid ; nor have I

failed to find it in specimens that I knew to be from an ovarian tumor." But so many of the best gynæcologists doubt the accuracy of his conclusions, it is but fair to say that the question is far from being settled.

As to the treatment of these tumors he says: "It is not necessary to interfere, in any manner, with some cases of ovarian dropsy. Indeed, it is right to let all cases alone that do not impair the health or threaten the life of the patient. There are many cases which advance slowly, or remain stationary for a great many years, and prove but an inconvenience. We would not be justified in active interference in these cases; much less should we do anything directly for cases in which complications of a fatal character exist, e. g., phthisis or cancer, albuminuria, etc.

"When, however, the disease is making obvious progress, and particularly when the advance is sufficiently rapid to leave but little doubt of its proving fatal within the average time of their duration, we are bound to make every effort within our power to save, or prolong as much as possible, the life of our patient."

The treatment is described as palliative and curative.

The dangers of tapping are fully described, and the means of avoiding them plainly pointed out, "but the dangers and benefits of tapping cannot and ought not to be estimated by comparison with other operations. Each operation, of whatever kind, has its place, and is followed by its good or bad effects, for the reason, among others, that it is appropriate or inappropriate."

Our author believes, with almost all authorities, that the curative treatment of ovarian cystomata is practicable only by surgical means, and while many of the plans, by medical agents and otherwise, which have been recommended, are described, they are interesting simply as facts of history.

Several surgical procedures are described for curing the disease beside complete removal. One is pressure. "After tapping the cyst or cysts, apply firm and continued pressure, as much as the patient and the abdominal organs can bear, to excite a moderate degree of inflammation in the collapsed cyst, and in this way obliterate its cavity, a plan of treatment frequently successfully adopted by Baker Brown, of London. This plan, when carefully managed and watched, is considered better than any other, except the complete extirpation of the ovary.

Another plan is to evacuate the sac and then inject some irritating substance, as iodine, into the nearly emptied cyst, to incite inflammation which shall obliterate the cavity.

The author suggests that alcohol, brandy, or some other local stimulant whose effect after absorption would be more transient as well as less powerful, would perhaps answer just as well as iodine.

The plan of making a fistulous opening, communicating with the peritoneal cavity, or the external surface directly, or indirectly through the vagina or rectum, is doubtless the most effectual plan for obliterating the sac, and, at the same time, the most dangerous, as a large number of cases result fatally—though numerous cases of cures by this method are reported.

Electrolysis, too, has its advocates, who report many cures and some deaths. But this method has not been sufficiently tried as yet, for one to judge of its results or determine the kind of cases most likely to be benefited by it.

The vaginal method of ovariotomy is described and recommended in suitable cases.

More than forty pages are devoted to the full description of abdominal ovariotomy, including the treatment of the pedicle, the ligature, drainage, the complication of pregnancy, the preparation of the patient for the operation, the steps in the operation, the accidents during the operation, and the after treatment, including the care of the patient, the wound and the complications, such as shock, hæmorrhage, peritonitis and septicæmia, closing with personal statistics. Dr. Byford uses the double-plated silver ligature, cutting it short and dropping the pedicle into the pelvis, and then carefully closing the abdominal wound.

Of the antiseptic treatment he says: "The value of this method, during the operation and after-treatment, is now so well established that I do not consider it necessary to do more than to express my concurrence in its use, and insist that no ovariotomy should be performed without it."

In the preparation of the patient all her functions are to be in the best possible condition, especially the skin, kidneys and alimentary canal. "If the patient is greatly depressed by recent bereavement or other cause of intense grief, the indications should be very urgent to justify the immediate removal of the tumor."

If the patient menstruates, he would operate soon after the menstrual flow.

"The personal preparation of the surgeon, assistants and attendants shonld be carefully made. Perfect cleanliness in them is a matter of paramount importance; to this end, ablution of the hands and cleansing of the nails must be thorough immediately preceding the operation; and all articles used in the operation should be as clean as possible."

Æther is always used as the anæsthetic. "All the sponges used should be soft, free from sand and thoroughly carbolized. The carbolized water is from two to three per cent." It is interesting to note here in passing, that Mr. Keith, though reported to have abandoned the spray for fear of poisoning himself and his patients, has now returned to the weaker spray.

The full and accurate description of the steps of the operation cannot be epitomized, but must be studied in the work itself.

If adhesions are very numerous and firm, the method by enucleation, as taught by Prof. Miner, of Buffalo, is recommended.

After cleansing carefully and thoroughly the abdominal cavity, the carbolized spray is directed into the cavity, an assistant raising the lax abdominal walls, so as to perfectly carbolize every part of it.

"The incision is then closed by fine silk sutures and covered with patent lint, saturated with carbolized oil.

"The wound, thus covered, is further protected by cotton-batting five or six inches thick, which extends over the whole abdomen and down well upon the symphysis.

"The whole is secured by a flannel binder, from the pubes to the ensiform cartilage. The spray is continued until the oiled lint is placed over the wound."

In the after-treatment anodynes are recommended in such doses as are necessary to keep the patient free from pain, and no more. And the temperature of the room, in cold weather, is not allowed to rise above 60° Fah.

But it is impossible to give an abstract of chapters like the "Accidents during the operation," and the "After-treatment of ovariotomy." They must be studied carefully by any one who would attempt the operation. Short chapters on the diseases of the fallopian tubes, coccygodynia or coccyalgia, and electricity, complete the volume.

The proof reading has evidently been hurried or careless. On page 33, Skene is spelled "Skein," and Reynolds "Renolds." Page 143, "Capelo" should read Tupelo; page 179, "perspirations" for perspiration; page 233, "boject" for object; page 242, "is avoided" for is to be avoided; page 277, "Spielberg" for "Spiegelberg; and the same page, "velous" for villous; page 357, "Goupel" for Goupil, and probably "Bennet" for Bernutz; page 353, "pelvic" tumors '*is*'" for "*are*"; page 397, "Gerring's" for Gehrung's; page 462, "Siemond's" for Simon's; page 458, "reply" for rely; page 559, "Weir" Mitchell for Wier Mitchell; page 533, "Chasignac" for Chossaignac; page 561, "even in the more puerpural condition" for even more in the puerpural condition; page 597, and the corpuscles described by Drysdale for "and *none* of the corpuscles," etc.; page 609, "pubis" for pubes; and same page, "Dieulefois" for Dieulafoy; page 640, "grooved directory" for grooved director; and page 623, the sentence "and the patient escapes the annoyance and dangers which attend the great development that necessarily follows, especially when the operator is an experienced gynecological surgeon," is certainly ambiguous.

But a new edition must soon be demanded, and then we may expect these errors will be corrected.

The style is clear but concise, and always practical, and shows the author to be, as we all know, an experienced teacher.

The work as a whole is most valuable, both to the student and the practitioner, and probably better than any other single work upon gynecology—and there are now so many—represents American gynecological practice. D. T. N.

ARTICLE IX.—NITRO-GLYCERINE AS A REMEDY FOR ANGINA PECTORIS. By WILLIAM MURRELL, M.D., M.R.C.P., Senior Physician to the Royal Hospital for Diseases of the Chest, etc. Geo. S. Davis. Detroit. 1882.

After a short historical sketch of nitro-glycerine and the earlier experiments made in its internal administration by Mr. A. G. Field, of Boston, and Drs. Harley and Fuller, of London, whose statements quite conflicted with each other, the author gives his personal experience with the drug when taken by himself and administered to others in a state of perfect health.

These experiments, in the main, confirmed the observations of Mr. Field, and led the author to try the drug as a remedy for angina pectoris. He states that he has given it in several hundred cases, twelve of which are here placed on record.

Of these, four are classed as successful, and eight as unsuccessful, but in most of the latter the remedy seems to have given more or less relief for a time. In the four successful cases the remedy acted promptly, and gave very satisfactory results.

He usually began the treatment with a one per cent. solution of nitro-glycerine in alcohol, one minim of which was given three or four times a day in a tablespoonful of water. Unless the physiological effects, *i. e.*, headache, pulsations in the head, and drowsiness, were then marked, the dose was increased to two or four minims of the same solution every three or four hours, and an extra dose whenever an attack came on. The dose in most cases was subsequently rapidly increased to ten, fifteen, or twenty-five minims, and in a few cases to much more than this. It was found that sometimes the large dose produced less discomfort in the head than the smaller doses.

One minim of this solution, which is equivalent to gr. $\frac{1}{100}$ of nitro-glycerine, he considers a safe dose, and he thinks it always safe to rapidly increase the dose until its physiological effects are developed. Fifteen or twenty minims would usually be a poisonous dose, and in some cases, especially in women, he found it impossible to give more than half or even one-sixth of a minim of this one per cent. solution. Notwithstanding the very unpleasant results sometimes obtained from small doses, in one case he gave as much as one hundred and ten minims of this solution eight times a day with beneficial results. The author prefers the solution to other preparations of the remedy, though he has also used it in pills and tablets, the latter of which he says "are not very nasty."

A sufficient number of cases have been here recorded to illustrate Dr. Murrell's method of giving the remedy, and the good and bad effects which sometimes follow; but we regret that out of "several hundred" cases he has only given us the results in twelve, and has left us to draw our own conclusions with regard to "several hundred" others. Of these twelve cases, only four were successful, and one of these the doctor never saw. Eight were only partially successful; some of these seem not to have had the affection at all, and one had taken the remedy entirely on his own responsibility, and had not been under the author's observation any of the time. What became of the several hundred others? Let us hope they were cured at once, so that they were not obliged to return for treatment. E. F. I.

ARTICLE X.—THE TRANSACTIONS OF THE AMERICAN MEDICAL ASSOCIATION. Vol. XXXII. 1881. 8vo. cloth. pp. 684. Philadelphia: printed for the Association, 1881.

The first part of these consists of reports, and the address of the late President, Dr. John T. Hodgen, which is a short and practical dissertation on surgery. In the Section on Practice of Medicine, Materia Medica, and Physiology, the usual amount of trash is fairly represented. Dr. W. C. Wile in his article on Blood-Letting in Pneumonia, fails to tell us which disease he refers to, the croupous or the catarrhal pneumonia. As his experience related only to the last few months, it could not be of much value, and as his thirteen cases may have included some cases of catarrhal pneumonia, it is hard telling how beneficial the blood-letting might have been, since quinine and morphine were also used together with it. The next paper is an ordinary note, of no intrinsic value. A paper of Dr. I. E. Atkinson, of Maryland, "May Iodide of Potassium excite Bright's Disease?" is a valuable one for reference.

The next paper in order, Soluble Compressed Pellets, is a modest and disguised advertisement. Considering the commonplace of what precedes, it is refreshing that the next article be one on "The Materia Medica of the Future," by F. E. Stewart, Ph. G., of New York; unfortunately the realization of harmony between the patent medicine man and the ethical physician, must be a thing of the distant future indeed.

Is Croupous Pneumonia a Zymotic Disease? by Dr. D. W. Prentiss, of Washington, should be read by every old practitioner for his advantage. The section on Ophthalmology, etc., comes next.

The address on Obstetrics by Dr. Chadwick, of Massachusetts, has much statistical value, and contains a certain amount of information which must have cost its author a great deal of patient labor.

The next of the articles in that section are short, but valuable. The section on State Medicine is well filled; that on Diseases of Children opens with a paper of Dr. Jacobi on Contagious Diseases, which is of the greatest practical value, in regard to diseases of the adult as well. The section on Surgery is represented by many of the best surgeons in the country. The rest of the volume (464–684) includes Reports of Special Committees, Index to American Medical Necrology, By-Laws, The Code of Medical Ethics, a List of Permanent Members and the Index.

On the whole, these transactions have a good deal of intrinsic value, but if we desire the future *Journal* of the Association to prove a financial success, we will need to increase this intrinsic value ten fold in the near future. H. D. V.

Article XI.—A Text-Book of Human Physiology. By Austin Flint, Jr., M. D. Illustrated by three lithographic plates, and 315 wood cuts. Third edition, revised and corrected. 8vo. cloth, pp. 978 $6.00. New York: D. Appleton & Co. 1881. Chicago: Jansen, McClurg & Co.

Few authors have given so much time to the study of physiology as Austin Flint, Jr., and his larger work on the Physiology of Man is cyclopædic in character, the one under consideration being more of a compendium, although so voluminous. This book opens with a consideration of the blood, which represents the knowledge accumulated by many reliable observers, and that is a part of the value of the work, that every investigator receives credit for his discoveries. However, there is a deficiency in the discussion of the latest discoveries and views, regarding leucocytes, fibrin, red corpuscles, etc., and references are never made to the various works of the authors cited, and hardly any note is to be found at the bottom of pages as in Dalton or Foster.

Respiration is the next part considered, and satisfactorily. Then follow in order, Alimentation, Digestion, Absorption, Secretion, Excretion, Movements, Speech, Nervous System, Special Senses and Generation. This last is not treated of in as scientific a manner as the late discoveries demand; and we may add that although the work professes to give all valuable information on any subject it is not really progressive in character, and is not based as it should be on biology, but rather on long accumulated data.

As to the following mooted questions, the author teaches that we see objects inverted, that the blood is the cause of the contraction of the heart, that olfaction takes place from the contact of material emanations with the olfactory membranes, etc. As we might expect of so conservative a work, the various mental processes are not considered as physiological functions of the brain, though the subject is not entirely overlooked.

Before closing, we may state that while in many respects Flint's Text-Book is most complete, in many it is deficient, and in some rather antiquated; while the lack of proper references suggests that his larger work has the one just reviewed for an introduction.

Our intention would be misinterpreted if the preceding criticism deterred students from the use of the book, for it is certainly one of the best ever written in this country, and contains a great deal of valuable information. It is also printed and illustrated in very good style.

H. D. V.

ARTICLE XII.—A TREATISE ON HUMAN PHYSIOLOGY. By JOHN C. DALTON, M. D. Seventh edition, with 252 Illustrations. 8vo. half Russia, pp. 722. $7.00. Philadelphia: H. C. Lea's Son & Co. 1881. Chicago: Jansen, McClurg & Co.

This is the "old reliable," the American student's friend. The amount of matter contained in Dalton's is not more than half that found in Flint's, but the original work done by the author, the selection of material, the rapid succession of new editions, and the progressive views assimilated have all contributed to the success of this treatise. Another peculiarity of the book is that instead of adding new discoveries to obsolete views, the author has at each edition, cut the latter while adding to the former, so that the science of physiology becomes very simple, and comprehensive at the same time. The first 130 pages are consecrated to Physiological Chemistry, which is a new science appropriately introdnced in this place, as it is not yet made a special department of Medicine. Section II comprises the Functions of Nutrition. Section III, the Nervous System, and Section IV, Reproduction. That is a clear division and grouping, of a nature to greatly facilitate the study of a subject rather intricate. Reproduction is treated of in a remarkably scientific and simple manner, though partaking of general animal and vegetable physiology. Digestion receives a most practical treatment, and the Physiology of the Mind is very suggestive of new discoveries. On the whole, this is an excellent manual, a little costly for the amount of matter it contains, yet having the advantage of a large type, and the greater advantage of being the production of a master. H. D. V.

ARTICLE XIII.—A PRACTICAL TREATISE ON THE DISEASES OF CHILDREN. By J. F. MEIGS, M. D., LL. D. Seventh Edition, revised and enlarged. 8vo. cloth, pp. 1055. $6.00. Philadelphia: P. Blakiston, Son & Co. 1882.

This is one of the most voluminous works on the subject, and also one of the oldest. This edition differs from the preceding in many particulars and probably make it such as to compete successfully with the many newer books now in the market. The new article on food was greatly needed, and one would desire to have more of the medical rules under which children should be bred extensively described. The common diseases icterus, neonatorum and ordinary jaundice are not noticed, nor is epilepsy, nor headache. Diseases of the skin are treated of at some length, but eye and ear diseases are overlooked. As we should expect, the authors confound catarrhal fever, lung fever, and pneumonia, and call it a disease of the lungs, while most recent authors recognize a catarrhal pneumonia to be a lung disease; and a croupous pneumonia, an infectious disease. (See Ziemssen). This book is also liable to another criticism which applies equally well to other books whose first edition date of long ago, too large a part of the volume is taken to denounce old modes of treatment, by bleeding, tartar emetic, violent purgatives, etc., of which we had known nothing but for those useless discussions, which take the place of more valuable advice in the methodical treatment of disease. As to that matter of treatment we are rather surprised that the authors give so much prominence to opium in diarrhœas, and do not ever mention the valuable hydrarg. c. creta. But the treatment generally advocated through the book comprises the latest discoveries, and is fully up to the best works on therapeutics. Considering the amount of information contained, the book is rather cheap, and deserves a wide circulation. H. D. V.

ARTICLE XIV.—FROZEN SECTIONS OF A CHILD. By THOMAS DWIGHT, M. D. FIFTEEN DRAWINGS FROM NATURE, by H. P. QUINCY, M. D. Large 8vo. cloth, pp. 66.

These sections were made at Harvard, where the originals are preserved, hardened in alcohol. The author gives the following directions for making frozen sections: "First, be very sure that the body, or part to be frozen is in precisely the position you desire, and that there are no folds or indentations in the skin. I always use natural cold when possible. Weather much above zero (Fahrenheit) is unsatisfactory; but if the part is thoroughly chilled by several days exposure to a pretty low temperature, a night of 10° may possibly finish it. The body should be frozen like a rock—so much so that the operator cannot tell whether he is cutting bone or muscle. The sections should be made in a cold room, with a very sharp saw that has been chilled. When a section is cut its surface is obscured by a thick half-frozen saw dust, which is doubly thick if the freezing is not quite sufficient. It is wisest if time allows, to remove this at once, which is done by pouring a little hot water over the section, and brushing or scraping it off rapidly and carefully. If it is to be kept (the specimen) it should be laid on a piece of glass or wood, and placed at once while still frozen, in cold alcohol."

This essay is a model in its line, and could hardly be surpassed in excellence, typographical as well as anatomical. We should recommend the process in comparative anatomy, which, of late, has received so much attention. The topography of internal organs could not be more forcibly impressed on the student than through such plates. One would naturally desire that the head had been represented in a few sections, and so the extremities.

H. D. V.

Article XV.—A Rational Materialistic Definition of Insanity and Imbecility, with the Medical Jurisprudence of Legal Criminality, Founded upon Physiological, Psychological and Clinical Observations. By Henry Howard, m. r. c. s., Eng. 8vo, cloth, pp. 145. Montreal: Dawson Bros. 1882.

This work is really valuable, and will be welcomed in a time when the attention of every practitioner is turned toward insanity. It consists of a chapter, What is Insanity? one on the Medical Jurisprudence of Crime and Insanity, Criminal Responsibility, of a paper on the Brains of Criminals, by W. Osler, m. d., m. r. c. p., the Medico-Relations of Epilepsy, by James G. Kiernan, m. d., of Chicago; and "Some Experiences of a Barrister's Life," by Mr. Sergeant Ballentine; the whole hardly justifying the bombastic title, "The Philosophy of Insanity." This ought not to detract from a careful study of the book, however, as we are persuaded that much useful knowledge will be the result.

H. D. V.

ARTICLE XVI.—THE PRINCIPLES AND PRACTICE OF SURGERY. Being a Treatise on Surgical Diseases and Injuries. By D. HAYES AGNEW, M.D., LL.D. Profusely illustrated. Large 8vo., cloth, pp. 1066. Philadelphia: J. B. Lippincott & Co.

More than a notice of this 2d volume could hardly be expected. It is large and comprehensive, and suffices for all the ordinary purposes of surgeons. It will be, when completed, one of the largest works on surgery ever written by a single author, but will receive a serious competition from the cyclopœdias now being published on the same subject. We think we are justified in recommending smaller books to students, although valuable manuals are hardly to be found in the country. Agnew's Surgery will answer also as a reference book, and is quite an ornament on the shelf.

ARTICLE XVII.—A SYSTEM OF SURGERY, THEORETICAL AND PRACTICAL. In Treatises by Various Authors. Edited by T. Holmes, Surgeon. Edited in this Country by John H. Packard, A. M., M. D. Vol. III. 8vo. half Russia, pp. 1059. Philadelphia: H. C. LEA'S Son & Co. 1882.

This ends the series, and conforms in general with the two first volumes already received. It contains the diseases of the respiratory orgars; of the bones, joints and muscles; orthopœdic surgery; diseases and injuries to the nerves; gunshot wounds; operative and minor surgery; diseases of the skin, etc. Like the other volumes, this is well illustrated.

---

YELLOW FEVER.—Acting-Collector Goodrich telegraphed from Brownsville, Texas, as follows: The first case of yellow fever occurred in Matamoras a month ago (about July 12), introduced through Bagdad, Mexico, by railroad tramps from Tampico. Average daily deaths in Matamoras the past ten days, ten. The disease appeared in Brownsville two weeks ago. Total number of deaths, ten or twelve. August 12, 20 new cases of fever, and August 18, 32 new cases were reported. August 28, there were 46 new cases.

